# Incidence, etiology, sociodemographic and clinical characterization of acute respiratory failure in pediatric patients at a high-altitude city: A multicenter cohort study

**DOI:** 10.3389/fped.2022.1009375

**Published:** 2022-12-15

**Authors:** Sarha M. Vargas Muñoz, Sara De Vivero Haddad, Aldo M. Beltran, Carolina Bonilla Gonzalez, Melisa Naranjo Vanegas, Sergio Moreno-Lopez, Paola Rueda-Guevara, Pedro Barrera, Juan Gabriel Piñeros, Luz Marina Mejía, María Lucia Mesa, Sonia Restrepo-Gualteros, Olga Lucía Baquero Castañeda, Andrea Ramírez Varela

**Affiliations:** ^1^ Department of Pediatrics, Universidad de los Andes, Medical School, Bogotá, Colombia; ^2^ Pediatric Intensive Care Unit, Fundación Santa Fe de Bogotá University, Bogotá, Colombia; ^3^ Department of Public Health, Faculty of Science, Universidad de los Andes, Bogotá, Colombia; ^4^ Pediatric Intensive Care Unit, Medical Epidemiologist, Fundación Santa Fe de Bogotá University, Bogotá, United States, Colombia; ^5^ Department of Pediatrics FARA Group, Bogotá, Colombia; ^6^ PDH Epidemiology, Bogotá, Colombia

**Keywords:** acute respiratory failure (ARF), high altitude, pediatric cohort study, pediatric critical care, multicenter study design

## Abstract

**Background:**

Acute respiratory failure is a life-threatening medical condition, associated with a variety of conditions and risk factors, including acute respiratory diseases which are a frequent cause of pediatric morbidity and mortality worldwide. In Colombia, the literature related to ARF is scarce.

**Objective:**

To determine the incidence, causes, and sociodemographic and clinical characteristics of ARF in three hospitals in Bogota, a high-altitude city located in Colombia, during the COVID-19 pandemic.

**Methods:**

A multicenter prospective cohort study called the FARA cohort was developed between April 2020 – December 2021. Patients older than one month and younger than 18 years with respiratory distress who developed ARF were included.

**Results:**

685 patients with respiratory distress were recruited in 21 months. The incidence density of ARF was found to be 41.7 cases per 100 person-year CI 95%, (37.3–47.7). The median age was 4.5 years.. Most of the patients consulted during the first 72 h after the onset of symptoms. Upon admission, 67.2% were potentially unstable. The most frequent pathologies were asthma, bronchiolitis, pneumonia, and sepsis. At admission, 75.6% of the patients required different oxygen delivery systems, 29,5% a low-flow oxygen system, 36,8% a high-flow oxygen system, and 9,28% invasive mechanical ventilation. *SARS-COV-2, respiratory syncytial virus, rhinovirus/enterovirus, and adenovirus* were the most frequently isolated viral agents. The coinfection cases were scarce.

**Conclusions:**

This multicenter study, the FARA cohort, developed at 2,600 meters above sea level, shows the first data on incidence, etiology, sociodemographic and clinical characterization in a pediatric population with ARF that also concurs with the COVID-19 pandemic. These results, not only have implications for public health but also contribute to the scientific and epidemiological literature on a disease developed at a high altitude.

## Introduction

Acute respiratory failure (ARF) is a life-threatening medical condition, characterized by increased preponderance for admission to pediatric intensive care units (PICUs), and frequently associated with the need for invasive mechanical ventilation (1–4). ARF is defined as the inability to maintain delivery of oxygen to tissue, but also as the inability to remove carbon dioxide from them (5). Epidemiology is not well described, due to inconsistent and heterogeneous diagnostic criteria (6). The incidence of ARF varies from 1.4 to 9.5 cases per 100,000 children and adolescents per year in the world, with estimated mortality over 24%–34% (6–8). Early diagnosis can be made by the physician who has a high index of suspicion, and who is aware of the clinical situations in which respiratory failure is likely to be a complication (9).

In Colombia, the literature related to the subject is scarce. The 2020 National Public Health Surveillance System-SIVIGILA (report from the Colombian National Health Institute – INS) and the National Demographics Department – DANE, mentioned the main cause of mortality in children under 5 years, which is given by acute respiratory infections with a mortality rate of 6.37 per 100,000 inhabitants, associated with ARF (10). By 2018, mortality from ARF was 2.52 cases per 100,000 children and the most common etiologic agent isolated among the previous 5 years was the Respiratory Syncytial Virus (RSV) (11, 12). During the year 2021, the highest proportion of emergency department hospitalizations due to ARF occurs in children aged 1 year with 20.1%, followed by children aged 2–4 years with 16.7% being COVID-19 one of the principal etiologies for ARF (13).

ARF is a generic term that encompasses a heterogeneous spectrum of diseases (5). There are multiple causes and risk factors for its development, microbiological, anatomical, social, demographic, exposure, nutritional, and hospital etiologies have been described (14–16). Careful assessment of history, complete physical examination, and evaluation of laboratory parameters may clarify the diagnosis (6). The recognition of patient's risk factors allows the institutions to provide an appropriate and timely management, and thus improve quality of life, and potentially reduce complications, severity in the course of the disease, and death (17).

Given the relevance of ARF in the world and the little information available in Colombia and Latin America, the aim of this research is to describe the causes, sociodemographic and clinical characteristics and estimate the ARF incidence of a pediatric population of three hospitals in a high-altitude city, contributing to the epidemiology and scientific literature with solid evidence.

## Methodology

### Study design

FARA is a multicentric cohort study conducted in three centers in Bogotá the capital city of Colombia, an upper-middle-income country. Bogota is located at a high altitude, 2,600 meters above sea level.

### Settings

The three centers included in the FARA cohort are described as follows. The first one, Hospital Universitario Fundación Santa Fe de Bogotá (HUFSFB) is a fourth-level of complexity health service institution which is located in the north of the city and it is a reference center in the country. The second one, Clínica Infantil Colsubsidio (CIC) is a pediatric hospital located in the center east of the city. The third institution, Instituto Roosevelt (IR), is in the center east of the city and has a large group of patients with a variety of neurologic conditions.

### Sample size

The main objective of the FARA study was to characterize ARF globally, so a sample size was calculated using a cumulative global incidence of ARF of 6.27%. The formula described by Peduzzi et al. was used (18), adjusting for a finite population (i.e., previous records of the total number of cases expected or that there were previous years in the institutions) to obtain a minimum necessary number of samples to make an association model where at least 3 covariables have a significant association with severity or mortality of ARF according to with literature (17). A simulation study of the number of events per variable in logistic regression analysis. The calculated sample size was 477 subjects with respiratory distress, with a sensitivity of 95% and a margin of error of 5%, and an additional 20% (18) was included considering losses and refusal of participation by eligible subjects.

Consecutive, non-probabilistic sampling was carried out for convenience once the eligible subjects were identified in the three institutions. This type of sampling was the best among the possible types of selection considering the objectives and design of the study. The number of individuals collected in each institution was evaluated bimonthly to monitor recruitment goals, considering the clinical history registers, and thus be able to intensify field collection strategies in institutions with a low collection rate.

### Participants

The study population included the pediatric population aged 1 month to less than 18 years old. Eligible participants were aged between 1 month to less than 18 years, had difficulty breathing defined as showing rapid breathing according to age (tachypnea), increased respiratory effort (retractions), or inadequate respiratory effort (slow/gasping breathing). Participants were enrolled in the three hospital's emergency departments, the pediatric intensive or intermediate care units, the general pediatric wards, or referred for hospitalization to one of these hospitals. ARF in our study was defined as the need for a high flow oxygen device or noninvasive/invasive mechanical ventilation. Some patients with respiratory distress but without ARF were identified and enrolled in the clinical history registry of each institution. The only exclusion criteria were an undergoing pregnancy. The patient´s legal guardian signed the informed consent, and patients 8 years of age or older, were asked to assent to participate in the study. It was considered as a requirement that either the patient or their legal guardian spoke Spanish or English. FARA study was approved by the HUFSFB's Corporate Research Ethics Committee, CIC's Bioethics Committee, and IR's Research Ethics Committee. Record: CCEI-11,899–2020.

Data collection was carried out at five points during the follow-up and each of them had a specific questionnaire, in the clinical scenery, that evaluated sociodemographic, clinical, and laboratory variables associated with individual characteristics of the patient and their disease process, but also, components of quality of life and the impact secondary ARF. To ensure study reproducibility, each time subjects were lost to follow-up at any time, new eligible participants were recruited consecutively until the defined sample was completed.

#### Point 0

Once the patient with respiratory distress was recruited, a preliminary record was made with the basic information of the patient, his clinical data, his personal and family history, as well as the oxygen device required at admission. If the patient developed ARF, he was followed up at 5 additional times.

#### Point 1

The first point is when the patient developed ARF. At this point, the basic data of the patient's registry, clinical data, medical history, and data taken at admission, including the type of oxygenation mechanism, were considered.

#### Point 2

In the second point, follow-up data was taken at 48 h after the patient developed ARF, including clinical data, ventilation parameters, evolution, and management at this moment.

#### Point 3

In the third point, data was taken at the patient's hospital discharge, including data on clinical evolution, indications for home, and discharge status (alive or dead).

#### Point 4

The fourth time was at 30 days post-discharge. Follow-up data was taken 30 days after the patient's hospital discharge. Quality of life was followed with two scales. If the patient was less than 8 years old, the ITQOL (Quality of life questionnaire) was applied. If the patient is more than 8 years old, the KIDSCREEN (Children quality of life questionnaire) was applied.

#### Point 5

The fifth time was at 60 days post-discharge. Follow-up data was taken 60 days after the patient's hospital discharge. Quality of life was followed with two scales. If the patient was less than 8 years old, the ITQOL (Quality of life questionnaire) was applied. If the patient is more than 8 years old, the KIDSCREEN (Children quality of life questionnaire) was applied.

**Figure 1:**
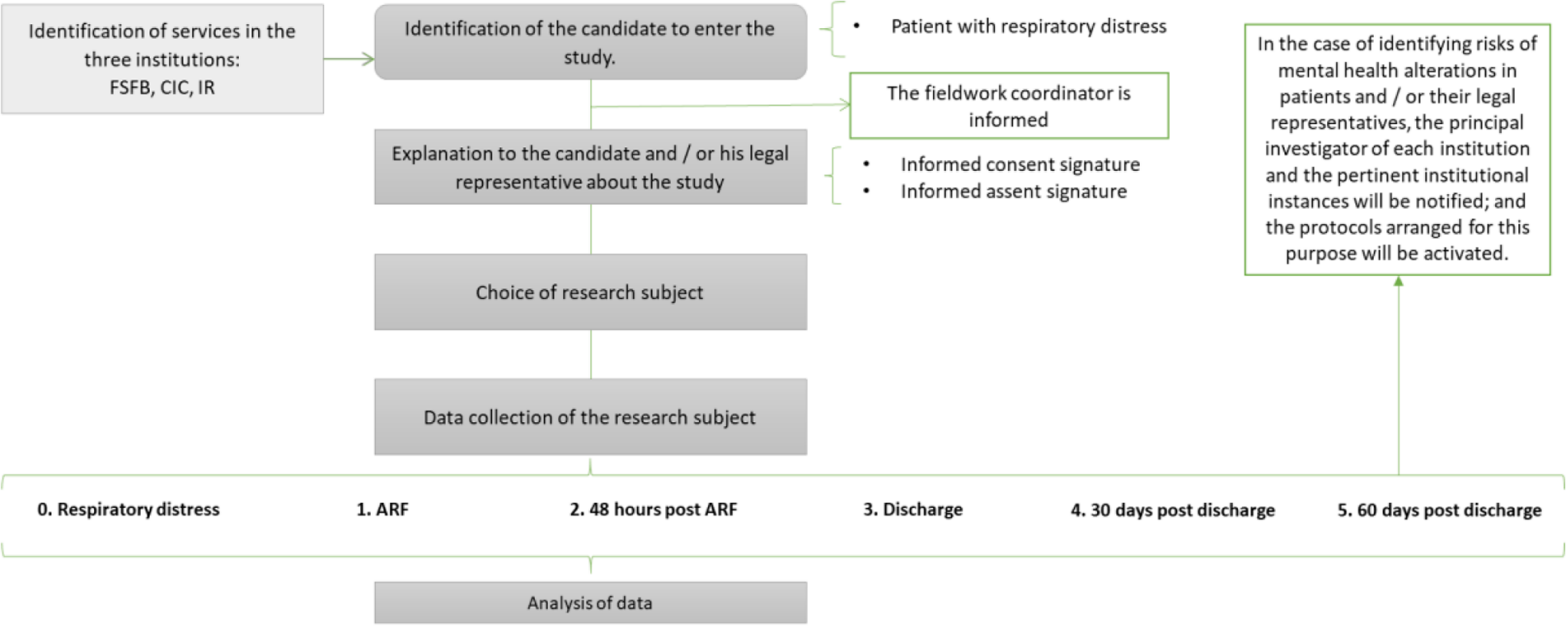
Flowchart of activities for each study participant.

**Figure 2:**
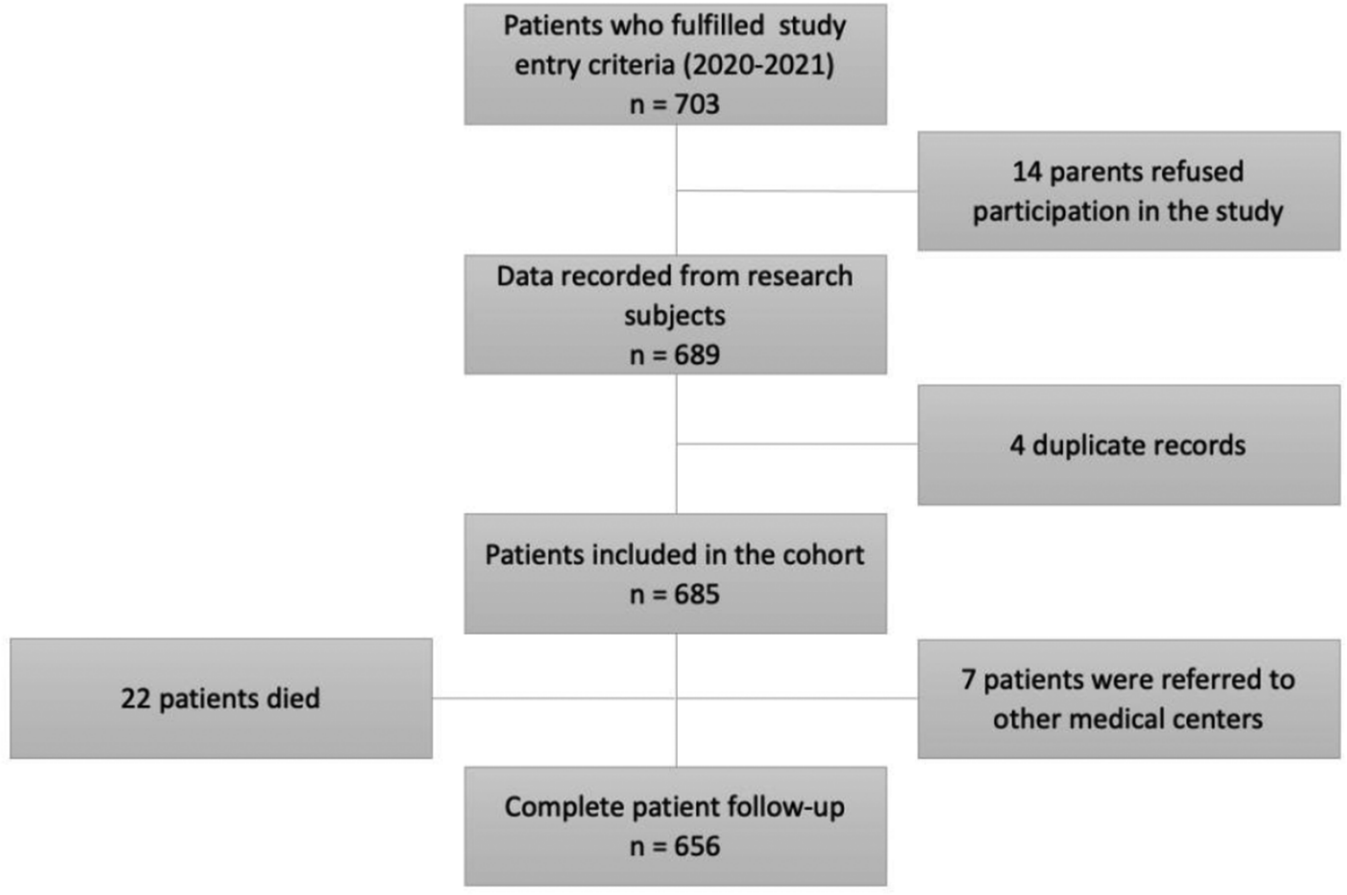
Flowchart of the number of patients followed.

Figure 1 summarizes the follow-up activities for each study participant and Figure 2 the numer of patients followed. Please feel free to check the contents of the questionnaires and data collection tools used in the different points by going to the Supplementary material.

**Table 1:** Sociodemographic characteristics of the ARF patients.

(a). General characteristics			
Characteristics	Total (*N*) 296	%	*p*-value**
Sex			0.138
Female	147	49.6	
Male	149	50.3	
Age			0.006
Infants (<2 years)	134	45.2	
Preschoolers (2–5 years)	74	25	
Schoolchildren (6–12 years)	53	17.9	
Teenagers (13–18 years)	35	11.8	
Anthropometric assessment (Z-score)			
0–5 years (*n *= 208)			
Severe acute malnutrition (<−3)	15	5.1	
Moderate acute malnutrition (<−2–≥ −3)	22	7.4	
Risk of acute malnutrition (≥–2–<–1)	31	10.5	
Appropriate weight for size (≥−1–≤+1)	87	29.4	
Risk of being overweight (>+1– ≤+2)	32	10.8	
Overweight (>+2– ≤+3)	19	6.1	
Obesity (>+3)	4	1.9	
>5 years (*n *= 88)			
Malnourished (<–2)	11	3.7	
Risk of malnourished (≥–2–<−1)	12	4	
Appropriate weight (≥–1–≤+1)	39	13.2	
Overweight (>+1–≤ +2)	17	6.1	
Obesity (>+2)	7	1.9	
Age			
Median	4,47 (5,00)	(0,47–7,43)	0.0448
Rural or urban residence			
Rural	45	15.2	
Urban	251	84.8	
Socioeconomic level			
1	51	17.2	
2	141	47.6	
3	79	26.7	
4	15	5.1	
5	7	2.4	
6	3	1	
Overcrowding			<0,00
Yes	47	15.8	
No	249	84.1	
Main caregiver			
Father/mother	289	97.6	
Brother/sister	1	0.3	
Grandfather/grandmother	5	1.7	
Main caregiver education			
None	6	2	
Primary	34	11.5	
Secondary	138	46.6	
Technical	69	23.3	
Professional	38	12.8	
Specialization	5	1.7	
Master's degree	6	2	

(b) Background of patients with ARF			
		Total (*N*) 296	%
Gestational age at delivery			
Preterm		71	24
Term		224	75.7
Post-term		1	0.3
Birth weight			
Low (<2,500)		67	23
Normal (>2,500–4,000)		176	60.4
Family illnesses			
Respiratory & Allergic disease		96	33.0
Cardiovascular diseases		36	12.2
Metabolic diseases		21	7.1
Oncological diseases		6	2.0
Neurological diseases		2	0.7
Gastrointestinal diseases		3	1.0
Autoimmune diseases		4	1.3
Rheumatological diseases		4	1.3
Comorbidities			
Yes		135	45.6
Respiratory disease		73	54
Neurological		43	31.8
Gastrointestinal		25	18.5
Genetic		22	16.3
Cardiovascular		11	8.1
Hematological		10	7.4
Renal		8	5.9
Allergies		8	5.9
Rheumatological		4	3
Endocrine		3	2.2
Hospitalizations			
In the last year			
1		51	17.2
2		19	6.4
3		8	2.7
4		5	1.7
5		2	0.7
None		211	71.3
Breastfeeding			
Exclusive		107	36.1
Absence		19	6.4
Not exclusive		170	57.4
Breastfeeding median time	(9.1 months)		
Environmental exposures			
Smokers		44	14.9
Pets		92	31.1
Schooled siblings		73	24.6
Contact with a person with respiratory symptoms		48	16.2
Immunizations (vaccines)			
Complete		263	88.8

ARF, Acute Respiratory Failure.

* Socioeconomic strata as defined by National Department of Statistics (DANE) of Colombia: 1 (very low) to 6 (high strata).

### Variables, data sources, and statistical methods

The development of acute respiratory failure was the primary outcome that was analyzed. Secondarily, the following were analyzed:

*Variables from the clinical history:* Background (pathological, gestational age at birth, birth weight, feeding with breastfeeding, previous hospitalizations for the same cause, vaccination status, physical examination on admission: vital signs, condition nutritional, respiratory evaluation, state of consciousness), risk exposure, schooled siblings, social, overcrowding, passive smoking, socioeconomic stratum. The anthropometric and nutritional classification was analyzed according to the growth and development tables for the Colombian pediatric population which are adopted from the growth patterns of the World Health Organization – WHO (19).

*Variables from the admission laboratories:* complete blood count (CBC), identification of viruses in the respiratory tract, identification of bacteria in the blood or respiratory tract, identification of another microorganism in the respiratory tract or blood. Among the institutions involved in the research, the HUFSFB routinely applies film array, the other institutions only in specific cases.

*Variables associated with diagnostic images:* chest *x*-ray, ultrasound, magnetic resonance imaging, chest tomography, among others.

Descriptive statistics were carried out for all the variables of the instrument collected; the quantitative variables were summarized using central tendency and dispersion measurements according to their distribution. Bivariate analyses were performed for sociodemographic and clinical variables. Categorical variables are described as frequencies and percentages and X2 tests or Fisheŕs exact test was compared. Continuous variables were presented as medians and interquartile ranges (IQR). Finally, the incidence density and mortality density rate were calculated for acute respiratory failure in our patients. For the density of incidence, a result was grouped by sex and age. For mortality density, it was calculated by sex and age.

All analyzes described above were performed with Stata Version 17 (StataCorp LLC, Texas, United States).

### Bias

The investigators followed a standardized protocol, inclusion, and exclusion criteria to avoid selection bias and confounders that threaten the internal validity of the study.

Different strategies were carried out to prevent possible biases in this type of study. They were aimed at preventing information bias, interviewer bias, volunteer participants, questionnaire bias, diagnostic suspicion bias, among others.

## Results

A cohort of 685 patients with respiratory distress and similar gender distribution (men 53.5% and women 46.4%); were recruited in 21 months. The incidence density of ARF was found to be 41.7 cases per 100 person-year IC: 95%, (37.3–47.7). The median age was 4.5 years; the majority were infants (45.3%), with an incidence density by sex of 47.8 cases per 100 person-year with an CI: 95%, (39.8–55.0) in the female group, and 37.7 cases per 100 person-year with an CI: 95%, (32.1–44.2) in males. Most of them came from urban areas (84.8%). The rest of the population came from rural areas**.** Regarding the socio-economic classification, 47.6% belonged to a low stratum (2 on a scale of 1–6 for the Colombian population) (20). The principal caregiver was the father/mother in 97.6%, most of them had basic education (58.1%).

About the perinatal background, most of the patients were born at term (75.7%) and had a normal birth weight (60.5%). The 33.0% had a first-degree family history of respiratory or allergic diseases (asthma, dermatitis, and rhinitis). Most of the children had normal weight, the malnourished population with ARF was 16.2% and the obese or overweight population was 15.9%. (Table 1). Almost half of the population had comorbidities, mainly associated with respiratory and allergic disease, principally asthma among others (54.1%), followed by neurological diseases, (31.8%).

Almost one-third had at least one hospitalization in the year before the recruitment (28.7%). Some children from the cohort had previously required a high-flow oxygen device as part of the management of respiratory diseases (10.8%), mainly high-flow oxygen cannula. Other children had required invasive mechanical ventilation in 16.5%. The previous results related to general characteristics and background are described in Table 1.

## Acute respiratory failure

The place with the highest frequency of ARF diagnoses was the pediatric intensive care unit of the three institutions. Most of the patients consulted during the first 72 h after the onset of symptoms. The median number of days of illness before hospital admission was 5.9 days and, 16.2% had contact with people with respiratory symptoms during the 15 days before the entrance. The main reason for consultation was related to respiratory symptoms and respiratory distress (70.9%) (Table 2).

**Table 2:** Clinical characteristics of the patients with ARF.

Characteristic	Total *N* = 296	%
Reason for consultation				
* *Fever			14	4.7
* *respiratory distress			138	46.6
* *Paleness/general discomfort			3	1.0
* *Respiratory symptoms			72	24.3
* *Seizures			12	4.0
Time until consultation				
* *Median		5.9		
* *≤72 h			214	69.2
* *>72 h			91	30.7
Initial Assessment				
* *Stable			64	21.6
* *Potentially unstable			201	67.9
* *Unstable			31	10.5
Microbiological isolation				
Virus				
* Respiratory syncytial virus (RSV)*			56	19.2
* Rhinovirus/Enterovirus (RV/EV)*			29	9.9
* SARS CoV 2*			66	23.2
* Adenovirus (ADV)*			13	4.5
* Influenza A (InfA)*			5	1.7
* Influenza B (InfB)*			7	2.4
* Coronavirus NL63 (CV NL63)*			1	0.3
* CoronavirusHKU1 (CV HKU1)*			1	0.3
* Parainfluenzae 1 (PIV1)*			1	0.3
* Parainfluenzae 3 (PIV3)*			1	0.3
Bacteria				
* S. aureus*			2	0.6
* E. coli*			1	0.3
* K. oxytoca*			1	0.3
* K. pneumoniae*			2	0.7
* S. agalactlae*			1	0.3
* S. hominis*			1	0.3
* S. pneumoniae*			1	0.3
* H. influenzae*			1	0.3
* Mycobacterium*			1	0.3
* Pseudomona aeruginosa*			1	0.3
Coinfection				
* *Coinfection virus─virus			18	6.1
* *Coinfection virus─bacteria			4	1.4
* *Coinfection bacteria─bacteria			2	0.7
Viral isolation by age (*n*)				

	≤2 years	2–5 years	6–12 years (*n*)	13–18 years (*n*)
*RSV*	45	9	4	2
*RV/EV*	11	13	7	–
*SARS-CoV-2*	33	18	7	8
*AV*	4	5	1	2
*Inf.A*	2	–	–	3
*Inf.B*	3	–	1	3
*CV-NL63*	1	–	–	–
*CV-HKU1*	1	–	–	–
*PIV 1*	1	–	–	–
*PIV 3*	1	–	–	–
***Total***				185

Characteristic	Total *N* = 296	%
Blood sample				
* *Anemia			49	16.7
Chest *x*-ray (*n* = 291)				
* *Inflammation			142	48.8
* *Alveolar occupation			79	27.1
* *Atelectasis			20	6.9
Initial ventilation device				
* *Cephalic mask (Hood)			1	0.3
* *Non-rebreather mask			101	34.2
* *Venturi system			15	51
* *High flow nasal cannula			142	47.9
* *Invasive mechanical ventilation			35	11.8
* *Non-invasive ventilation			2	0.7
Use of Antibiotics (*n* = 120)			120	41.2
* *Before the first 72 h			47	16.2

By the time of admission, according to the pediatric approach triangle (appearance, circulation, and work of breathing) (21), most of the population was potentially unstable or unstable (78.4%). Almost all the diagnoses were due to respiratory illness (81.8%). Hypoxic respiratory failure (type 1) was the commonest (82.1%). Asthma 27.0%, bronchiolitis 23.6%, pneumonia 12.8% and sepsis 10.2% had the highest rates of frequency (Table 3).

**Table 3:** Diagnosis of acute respiratory failure and classification of ARF.

	Total (*N*) 296	%
Respiratory system		
Bronchiolitis	70	23.6
Asthma	80	27.0
Pneumonia	38	12.8
Aspiration pneumonia	4	1.4
Chemical pneumonitis	1	0.3
Foreign body	3	1.0
Laryngotracheitis	10	3.4
Drowning	1	0.3
SLE/Alveolar hemorrhage	2	0.7
Vasculitis/pulmonary involvement	1	0.3
Tuberculosis	1	0.3
Pulmonary edema	1	0.3
High altitude pulmonary edema	1	0.3
Pulmonary embolism	1	0.3
Pulmonary hypertension	1	0.3
Central Nervous System		
Meningitis	3	1.0
Encephalitis	3	1.0
Status epilepticus	11	3.7
Guillain barre	1	0.3
POP CNS tumor resection	1	0.3
CNS tumor/Endocranial hypertension	4	1.4
Obstructive hydrocephalus	1	0.3
Spinal muscular atrophy	1	0.3
Type of respiratory failure		
Type 1 or hypoxic	243	82.1
Type 2 or hypercarbia	18	6.1
Type 3 or Mixed	26	8.8
Cardiovascular system		
Heart failure/Congenital heart disease	1	0.3
heart failure/Tetralogy of Fallot	1	0.3
POP closure VSD/Low cardiac output syndrome	2	0.7
heart failure/VSD	1	0.3
POP closure ASD + VSD/hypovolemic shock	1	0.3
Gastrointestinal system		
Sepsis of abdominal origin	4	1.4
POP laparotomy for intestinal obstruction	1	0.3
Endocrinological system		
Diabetic ketoacidosis	3	1.0
Renal System		
Chronic kidney failure	2	0.7
Hemolytic uremic syndrome	1	0.3
Hematological system		
Febrile neutropenia	2	0.7
Other POP states		
POP craniosynostosis/hypovolemic shock	2	0.7
POP craniosynostosis/anaphylactic shock	1	0.3
POP Scoliosis/hypovolemic shock	3	1.0
POP tonsil resection/hypovolemic shock	2	0.7
POP kidney transplant	1	0.3
Others		
Septicemia	26	8.8
Burned	1	0.3
Cardiopulmonary arrest	1	0.3

At the entrance, some children required immediate ventilatory system support as high-flow oxygen system devices in 82.0%, non-invasive ventilation in 0.7%, and invasive mechanical ventilation in 11.8% of the cases. The etiological isolation method used the most, were the viral panel 38.1%, SARSCOV-2 PCR 35.1%, and the respiratory film array 27.8%. Regarding etiological isolated agents, viral ones were the commonest, like *SARS-COV2 23.2%, respiratory syncytial virus (RSV)* 19.3%, *rhinovirus/enterovirus (RV/EV) 10.0%, adenovirus (ADV) 4.5%.* The viral isolation was more frequent in the infant's group (children under 2 years of age). The occurrence of isolation for each type of virus was different for each age group. (Table 2). We had a low rate of bacterial isolation. The 50.5% of the recruited population didńt have any etiological isolation. A low rate of children who received antibiotics before the first 72 h of hospitalization (16.1%). The mean length of stay for respiratory admissions was 10.3 days.

We had 22 deaths, of which six were attributed to sepsis/multi-organic failure (5 of them were related to SARS-COV-2), five patients due to pneumonia, three to bronchiolitis, and one death was related for each of the following diagnoses: asthma, cardiogenic shock, systemic erythematous lupus (SLE)/Pulmonary hemorrhage, chronic kidney failure, hemophagocytic lymphohistiocytosis, central nervous system tumor, meningitis, Duchenne muscular dystrophy. The mortality density rate was found to be 24.8 cases per 100 person-year, CI: 95% (16.3–27.6). We didńt have differences by sex. Infants (children <2 years old) had the highest density mortality rate.

## Discussion

ARF is a clinical condition that happens when the respiratory system fails to maintain its main function, which is the gas exchange (21). It is the result of progressive and sudden deterioration of respiratory and circulatory function during various diseases that without prompt and appropriate intervention, could be related to significant morbidity and mortality (22). Few studies describe and characterize a population with ARF. This study, belongs to the first multicenter prospective cohort that was developed at a high-altitude city in Colombia, the third country with the largest population in Latin America, after Brazil and Mexico (23) and also concur with the COVID-19 pandemic Era. The FARA study contributes to the description of ARF in the pediatric population as a contribution to the scientific and epidemiological literature.

As a common endpoint to multiple clinical conditions, the incidence of acute respiratory failure in the pediatric population is difficult to ascertain (24). After 21 months of recruitment, the incidence density of ARF was found to be 41.7 cases per 100 person-year IC: 95% (37.3–47.7). Since we didńt have a fixed cohort over time, it was not possible to compare this finding with other studies.

Our study showed a high frequency of ARF in the youngest patients, mainly those under 5 years (70.3%), with a median age of 4.5 years, and presented a similar distribution by sex. There are similar studies, that have found related data, a retrospective analysis using prospectively collected clinical data within the LARed Network ARF clinical registry, who described the epidemiological characteristics of the pediatric patients in 8 countries (Argentina, Bolivia, Brazil, Chile, Colombia, Costa Rica, Ecuador, and Uruguay), and found that the median age of the ARF population was 5 months with a similar distribution by sex males (25). There is another prospective study developed in pediatric patients between 1 month and 15 years with ARF in India, where the highest frequency of ARF was in children less than 5 years old (6).

We found some social and demographic characteristics that were similar for the whole ARF cohort, some of them are well recognized as risks factors for the development of illness, morbidity, and mortality in the pediatric population (26). It is important to note that this study was carried out in an urban area, so it was to be expected that most participants were from this area. Even though all institutions admitted patients from all socioeconomic strata, from the highest to the lowest (27, 28), it was found that the majority of patients with ARF had a low socioeconomic stratum (7, 28), all the patients who died during the study belonged to the low socioeconomic stratum, a finding that coincides with the literature, which shows that social and economic disparities represent a risk factor for developing ARF (27) and it is also related to higher mortality rates (29).

Also, the study population was exposed to environmental factors such as smoking (14.8%) and contact with pets (38.0%). It has been reported in the literature that these exposures are related to an increased susceptibility to developing respiratory diseases or exacerbating chronic allergic diseases (30), some people from the cohort had comorbidities like asthma, dermatitis, and rhinitis that may represent a risk factor for the development of ARF. Overcrowded conditions have been reported as a risk factor for respiratory illness (26), however, not all the population presented it (15.9%). Children from our study were not highly exposed to people with respiratory symptoms (16.2%).

It has been described that having a history of exclusive breastfeeding and an adequate nutritional status of the patient could be related to protective factors against the presentation of respiratory disease and its complications (17). Our frequency of exclusive breastfeeding coincided with the national census of nutritional evaluation of the pediatric population – ENSIN – (36.1%) (31), however, it is lower than the world ranges (43.0%) and lower than the goal proposed by the world health organization (WHO) for the year 2025 set at 50.0% (32). Malnourish has been associated with infections, mortality, and poor outcomes in hospitalized patients and those admitted to the pediatric intensive care unit (PICU) (33), 16.2% of the children from the cohort with ARF had some degree of malnutrition, but we didńt find any association with the ventilatory required method or with mortality. However, we did find that our rate remains above the national report established by the ENSIN 2015 (3.7%) and the world rates for the same year (5.0%) (31).

One of the health system interventions to control acute respiratory infections and their complications as ARF, is the early diagnosis and treatment of diseases (34). Since the study was developed in three different institutions with distinct medical care protocols, availability and access to resources, we expected variations in the therapeutic approach, use of diagnostic strategies for etiological isolation, and timely use of the different ventilatory support devices.

Many of our patients consulted before 72 h. Those who initially required high-flow support or advanced ventilation mechanisms, such as orotracheal intubation, coincide with a consultation time to the emergency room beyond 72 h after the onset of symptoms, it could be associated with a possible correlation between consultation time and worse outcomes and represent an important target for health systems and public health programs.

The most frequent reason for consultation was the increased work of breathing (46.6%), and, like other studies, additional nonspecific signs such as cyanosis, paleness, general malaise, vomiting, fever, abdominal pain, headache, or drowsiness were found in a lower proportion (28). Initial physical examination of patients with ARF should focus on overall appearance, vital signs, and the ABCs (5). During the primary evaluation (35), most of the population had some signs of clinical instability, reflected in respiratory effort or alteration in vital signs. In the secondary evaluation, we found that almost half of the patients had comorbidities that in most of the cases involved more than one system, mainly, respiratory, and neurologic; those conditions have been related to the development of ARF and mortality from these causes. In a study in the United States, Flori et al. show that more than half (58%) of patients with ARF and multiple organ failure died (16, 36).

About 1/3 of the patients had a previous hospital admission in the last year, mainly associated with respiratory disease. There is a retrospective cohort study developed in Western Australia, which mentioned that the strongest predictor of respiratory morbidity related to requiring hospitalization after age 3, is a medical history with one or more hospitalizations for ARF during childhood (37).

ARF can be associated with pulmonary or extrapulmonary causes. It is diagnosed when the patient develops hypoxemia or hypercarbia (5). Hypoxic respiratory failure (type 1) was the commonest type seen in 82.1% of patients. There are three major etiological categories described in the literature, intrinsic and acquired lung disease, airway disorders, and neuromuscular dysfunction. We had a high rate of pulmonary and airway causes such as asthma, bronchiolitis, and pneumonia. Pathophysiology could be explained by V/Q mismatching and gas diffusion impairment in the pulmonary causes, and a smaller radius of the airway of the children who had airway disorders. As we mentioned, non-pulmonary causes need also to be considered, as sepsis and neurologic disorders (seizures and neuromuscular diseases), we had a high rate of sepsis and our cohort presented a high frequency of neurological diseases as a cause of ARF (6).

The study was planned prior to the COVID-19 pandemic Era, with the aim of characterizing a population with ARF located at high altitude, however, the moment in which it was decided to start recruiting the population coincided with the presentation of a global event that represented the cause of multiple social, epidemiological, environmental and public health changes. Given the above, we expected changes in the usual microbiology circulation behavior and seasonal isolation.

Viral infections, like other studies, were the most frequent etiology isolation in preschoolers and schoolers ages (38). In the scientific literature, respiratory syncytial virus (RSV), influenza A virus (InfA), influenza B virus (InfB), parainfluenza (PIV1,2,3), and adenovirus (ADV) have been the most common causative viruses for acute respiratory infections, especially for lower respiratory tract illness (38–41). However, in our study the usual isolation change. The presence of the COVID-19 pandemic may have changed recruitment rates during the study, resulting in changes and variations in the usual seasonal microbiological circulation for our region in the context of ARF, which could represent one of the major valuable findings of the study. The main viral agents in order of frequency were SARS-COV2, RSV, RV/EV, ADV, and Inf A and B. As an important find, we had a high rate of RV/EV isolation, there is growing evidence that supports that this pathogen can contribute to significant respiratory disease and could be associated with morbidity, admission to the PICU, or need of mechanical ventilation (42, 43). Unlike other studies, as a prospective study conducted in Madagascar that reports human metapneumovirus (HMPV) infection in about 14% of children with ARF, we didńt have any isolation of HMPV (39), but we found another type of coronavirus as NL63 and HKU1 in a low-rate proportion, demonstrating the diversity of acute respiratory infections pathogens and the variations in the viral circulation for each region. The viral isolation behavior was different between age groups, being higher in children under 5 years of age, mainly in infants (<2 years). Also, the frequency of isolation of each virus varies according to the age group. While RSV was common in children under 2 years of age, COVID and influenza lead the isolations in adolescents (Table 3). Bacterial isolation and coinfection were unusual.

Some of the population had anemia and from this group, 82.0% required a high-flow ventilation delivery system upon admission, and 22.0% required invasive mechanical ventilation. It is known that one of the main determinants of DO2 (oxygen delivery) are hemoglobin levels, so anemia may be an important risk factor for a poor outcome in respiratory failure and the requirement of mechanical ventilation, but nevertheless, there are very limited scientific data available (44).

Regarding mortality, most children who died were infants under 2 years old, and more than half had comorbidities (Table 1). Our main cause of mortality was sepsis, followed by pneumonia, similar findings described in an acute hypoxemic respiratory failure cohort reporting mortalities of around 30% for these two conditions (45).

## Conclusion

We describe a multicenter cohort study developed at 2,600 meters above sea level, and show the first data on incidence, etiology, sociodemographic and clinical characterization in the pediatric population. These results have implications for public health, giving detailed information on ARF in the pediatric population, that could lead to preventive interventions. The data obtained in the present study represent a starting point for the generation of new scientific knowledge in pediatric ARF. Also, conducting this study during the COVID 19 pandemic allowed observing different aspects of ARF during an unprecedented event, and it helps to understand the impact of this pandemic on pediatric patients.

## Data Availability

The original contributions presented in the study are included in the article/**Supplementary material**, further inquiries can be directed to the corresponding author/s.
